# Viral infection, proliferation, and hyperplasia of Hofbauer cells and absence of inflammation characterize the placental pathology of fetuses with congenital Zika virus infection

**DOI:** 10.1007/s00404-017-4361-5

**Published:** 2017-04-11

**Authors:** David A. Schwartz

**Affiliations:** Department of Pathology, Medical College of Georgia, Augusta University, Augusta, Georgia

**Keywords:** Placenta, Zika, Infection, Microcephaly, Pathology, Virus

## Abstract

**Purpose:**

Attention is increasingly focused on the potential mechanism(s) for Zika virus infection to be transmitted from an infected mother to her fetus. This communication addresses current evidence for the role of the placenta in vertical transmission of the Zika virus.

**Methods:**

Placentas from second and third trimester fetuses with confirmed intrauterine Zika virus infection were examined with routine staining to determine the spectrum of pathologic changes. In addition, immunohistochemical staining for macrophages and nuclear proliferation antigens was performed. Viral localization was identified using RNA hybridization. These observations were combined with the recent published results of placental pathology to increase the strength of the pathology data. Results were correlated with published data from experimental studies of Zika virus infection in placental cells and chorionic villous explants.

**Results:**

Placentas from fetuses with congenital Zika virus infection are concordant in not having viral-induced placental inflammation. Special stains reveal proliferation and prominent hyperplasia of placental stromal macrophages, termed Hofbauer cells, in the chorionic villi of infected placentas. Zika virus infection is present in Hofbauer cells from second and third trimester placentas. Experimental studies and placentae from infected fetuses reveal that the spectrum of placental cell types infected with the Zika virus is broader during the first trimester than later in gestation.

**Conclusions:**

Inflammatory abnormalities of the placenta are not a component of vertical transmission of the Zika virus. The major placental response in second and third trimester transplacental Zika virus infection is proliferation and hyperplasia of Hofbauer cells, which also demonstrate viral infection.

## Introduction

Following many months of intensive collaborative research, during which pathology studies played a prominent role, on April 13th, 2016 the United States Centers for Disease Control (CDC) in Atlanta issued a statement that Zika virus (ZIKV) was a cause of microcephaly and other severe fetal brain defects [1]. Since then, data continue to accumulate defining the etiological relationship between Zika virus infection in occurring in pregnancy and the development of fetal microcephaly and other intrauterine fetal malformations [2]. Analysis of the spectrum of pathologic findings from autopsies of fetuses and infants with congenital Zika virus infection, together with neuroradiology studies, has been of critical importance in defining the central nervous system abnormalities caused by the virus [3, 4]. Rapid progress is continuing in expanding the spectrum of clinical findings which constitute the congenital Zika syndrome [2, 5]. It should not be surprising that pathology studies have served an important role in defining the relationship between Zika virus and fetal infection [6]. During previous outbreaks and epidemics of emerging infectious diseases, pathology has proven to be a valuable tool for the understanding of how microorganisms can cause disease [7–9]. Pathology data are frequently incorporated into clinical and epidemiological investigations, and may be useful in designing strategies to interrupt disease transmission, to develop therapeutic interventions, and to answer important questions about the pathogensis of disease.

The Zika virus is a TORCH agent (Toxoplasma, Others, Rubella, Cytomegalovirus, Herpes) [10]. It has several characteristics in common with most other TORCH agents—the mother is often asymptomatic or has mild illness, yet the agent can cross the placenta and cause severe harm including brain damage, or even death to the fetus [10, 11]. In the current global Zika epidemic, scientific investigation is becoming increasingly focused on the potential mechanism(s) for Zika virus to be transmitted from an infected mother to her fetus. The placenta has a protective role in inhibiting the transmission of infectious agents to the fetus based upon the separation of maternal and fetal vascular supplies, the trophoblastic barrier, and possibly the occurrence of a population of macrophages (Hofbauer cells) in the chorionic villous stroma. However, it can also serve a permissive function in the transmission of infectious agents to the fetus, either by passive diffusion or by active transport. To understand vertical transmission of Zika virus, the placenta is of paramount importance. The human placenta is the largest of fetal organs, and forms a selectively-permeable barrier between the maternal and fetal circulations up to the time of birth. There remains little doubt that the placenta is the key organ through which fetal Zika virus infection develops, and it is important that the mechanism(s) by which this process occurs be understood. Several very recent observations by the author from 12 second and third trimester human placentas of infants with congenital Zika virus syndrome and microcephaly, together with experimental human placental cell infections, have provided potential clues to help answer this problem.

## Results

### Inflammatory cell reaction is absent in Zika virus infection of the placenta

**Fig. 1:**
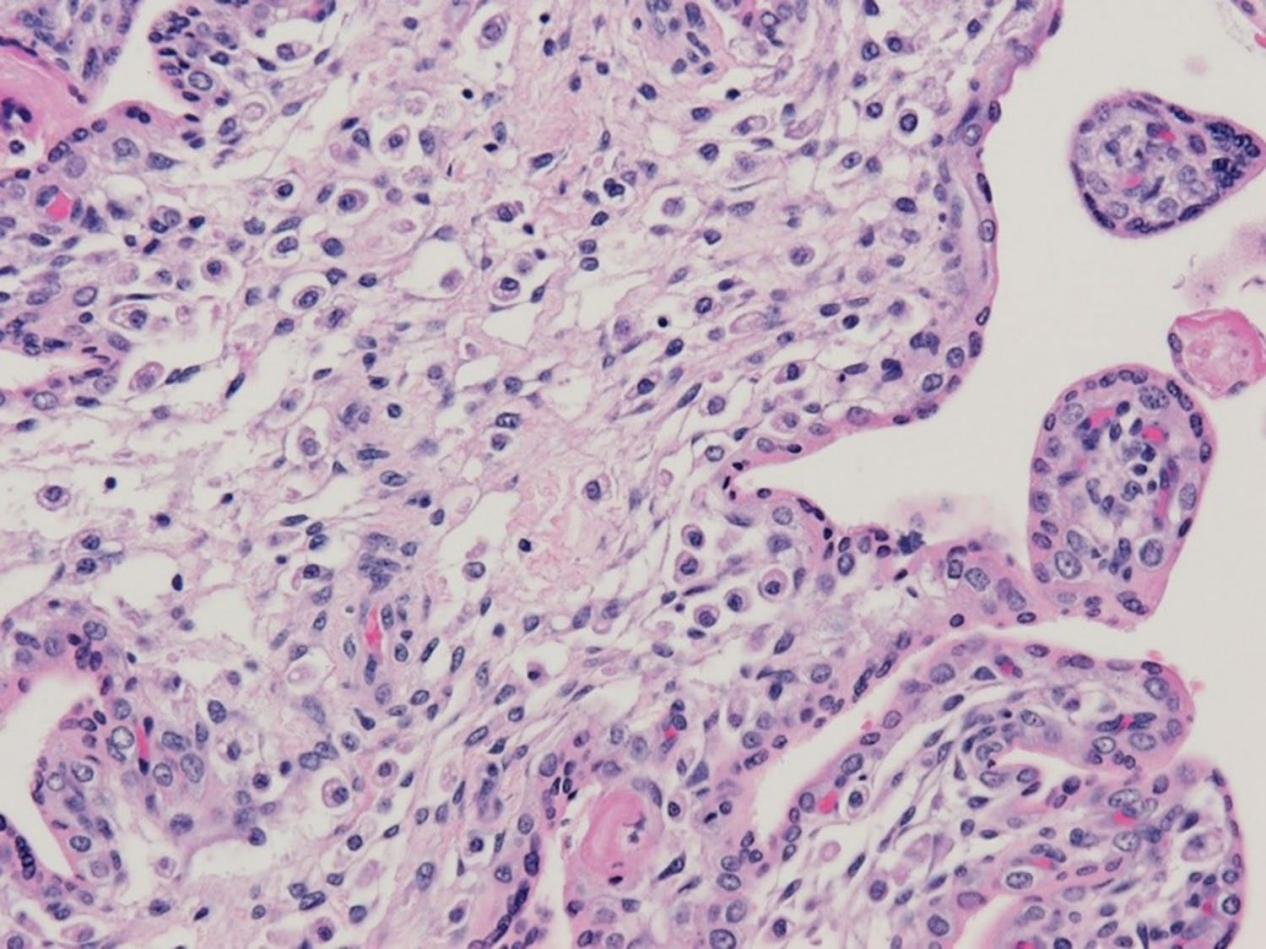
Appearance of chorionic villi from a stillborn infant at 32 weeks gestation with congenital Zika syndrome including microcephaly. The villi are greatly enlarged for gestational age and are hypercellular, but there is no villitis or necrosis. Most of the stromal cells are macrophages, termed Hofbauer cells. Hematoxylin and eosin stain

The histological hallmark in the placenta of most hematogenously transmitted vertical infections is villitis. When infectious agents pass from the maternal into the fetal circulation, they generally incite an inflammatory reaction in the chorionic villous tree of the placenta which is termed villitis. This is especially true for TORCH agents, such as syphilis, toxoplasmosis, cytomegalovirus, rubella, listeriosis, *Trypanosoma cruzi*, and others [10]. The villitides are categorized diagnostically based upon the predominant inflammatory cell type within the villi—acute (neutrophils), granulomatous (histiocytes and multinucleated giant cells), and chronic (lymphocytes and/or plasma cells) villitis. The inflammatory reaction which occurs in villitis can have cells originating in either the maternal blood stream (termed a maternal inflammatory response or MIR) or the fetal circulation (termed a fetal inflammatory response or FIR), or both. When villitis is found associated with villous necrosis, it is termed necrotizing villitis. An inflammatory reaction can also occur which is based between the chorionic villi in the intervillous space. This lesion is termed intervillositis, and is characteristic of *Listeria* infections. Examination of the second and third trimester placentas from fetuses having intrauterine infection with the Zika virus, together with recently reported observations, has determined that the inflammatory and necrotic lesions of the placenta which are typical of other TORCH infections do not occur in placentas from fetuses with intrauterine Zika virus infection (Fig. 1).

In contrast to maternal blood-borne infections entering the placenta, infections can also directly ascend from the vaginal canal, pass through the cervix, and reach the amniotic sac and placenta. This results in one or more inflammatory conditions of the placenta, termed chorioamnionitis (inflammation of the placental membranes), funisitis (inflammation involving the umbilical cord vessels), and deciduitis (inflammation involving the decidua, or endometrium)—examples include Group B streptococcus, *E. coli, Ureaplasma, Chlamydia, Mycoplasma*, and other vaginal microflora. Because the Zika virus is hematogenously transmitted to the fetus, chorioamnionitis and related abnormalities have not been associated with intrauterine infection by this virus.

The absence of villitis in transplacental Zika virus infection is unexpected, especially because the virus can cause necroinflammatory reactions when it reaches the fetal brain. Prior to the Zika virus epidemic, the most common hematogenously transmitted virus which did not produce villitis was the human immunodeficiency virus (HIV); however, this virus does not cause necroinflammatory damage to the fetal tissues.

### Transplacental Zika virus infection does not cause necrosis or scarring in the placenta

Those infectious agents which cause necrosis (irreversible cell death) in the fetus following vertical transmission typically also cause necrotic changes in the placenta. Infection or inflammation of the cells lining the blood vessels in the chorionic villi can result in endothelial necrosis, vascular obliteration, and thrombosis, with the production of avascular (scarred) villi. Infectious agents which induce villous necrosis include syphilis, cytomegalovirus, listeriosis, toxoplasmosis, and others. The Zika virus causes necrosis of cells in the fetal brain [3, 12]; however, analysis of placentas from fetuses with intrauterine Zika virus infection and microcephaly has not demonstrated viral-induced necrosis, either acute, subacute, or chronic. No necrosis of cells lining the villous capillaries and larger vessels (termed endotheliitis) occurs (Fig. 1). Because villitis and necrosis are not caused as a result of transplacental Zika virus passage, it is not surprising that villous scarring (fibrosis) from remote necrosis is also not found.

### Human placental stromal macrophages (Hofbauer cells) proliferate and increase in number in response to transplacental Zika virus infection

Hofbauer cells are fetal cells of monocytic origin and are a normal component of the stroma of the chorionic villi. They first appear in the chorionic villi at the 10th–18th days of gestation, and are believed to initially be of fetal mesenchymal origin, derived from monocyte progenitor cells of the hypoblast-derived yolk sac that have migrated to the mesenchymal core of the villi. As gestation progresses, it has been suggested that they are derived from a population of recruited fetal monocytes [13, 14]. Similar to macrophages in other organs, Hofbauer cells are large (10–30 µm diameter) cells with cytoplasmic processes which contain large vacuoles, pinocytotic vesicles, and intracytoplasmic granules. Their function includes phagocytosis of fluids and apoptotic materials, antigen presentation in response to infectious agents, and possibly an angiogenic role in early placental vasculogenesis, maintenance of placental water balance, and an endocrine function. Hofbauer cells have been characterized as M2/alternatively activated macrophages.

In normal pregnancies, Hofbauer cells diminish in number by the 4th–5th months of gestation, and can be difficult to identify without the use of antibody staining to macrophage antigens. Hyperplasia of Hofbauer cells is abnormal, and occurs in a wide variety of pathological conditions of pregnancy. These include villitis of unknown etiology (VUE), ascending infections, and maternal blood-borne infections which cause villitis such as TORCH infections including cytomegalovirus and syphilis [15, 16], and Chagas’ disease [17]. The mechanism by which Hofbauer cell hyperplasia occurs in response to an inciting factor is, at least in part, the result of proliferation of these cells within the chorionic villous stroma. Hofbauer cells can proliferate in response to various stimuli as demonstrated using antibodies to cellular proliferation markers such as Ki67, as well as the observation of mitotic figures in the villous stromal macrophages [18, 19].

**Fig. 2:**
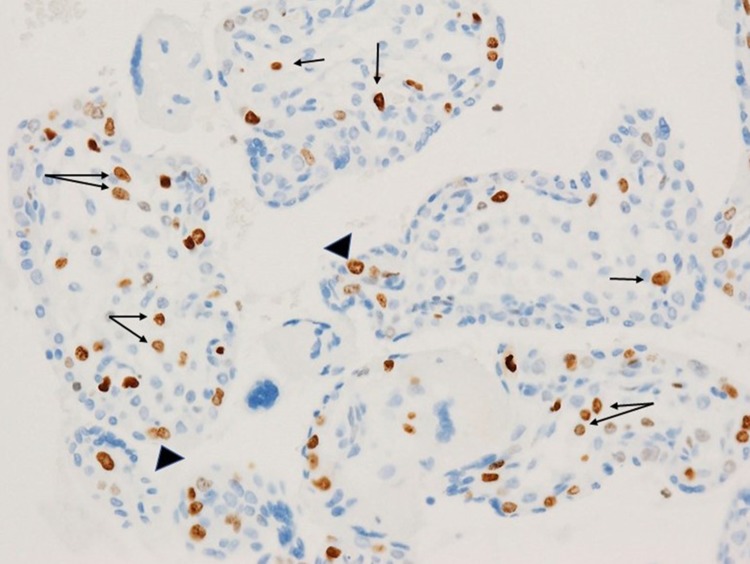
Proliferation of Hofbauer cells in a 21 week gestation Zika virus-infected placenta is evident by the nuclear staining of these macrophages using Ki67 (*arrows*), an antibody to a nuclear protein strictly associated with cellular proliferation. At the surface of the villus, occasional trophoblast cells (*arrowheads*) are also in the proliferation phase of the cell cycle

**Fig. 3:**
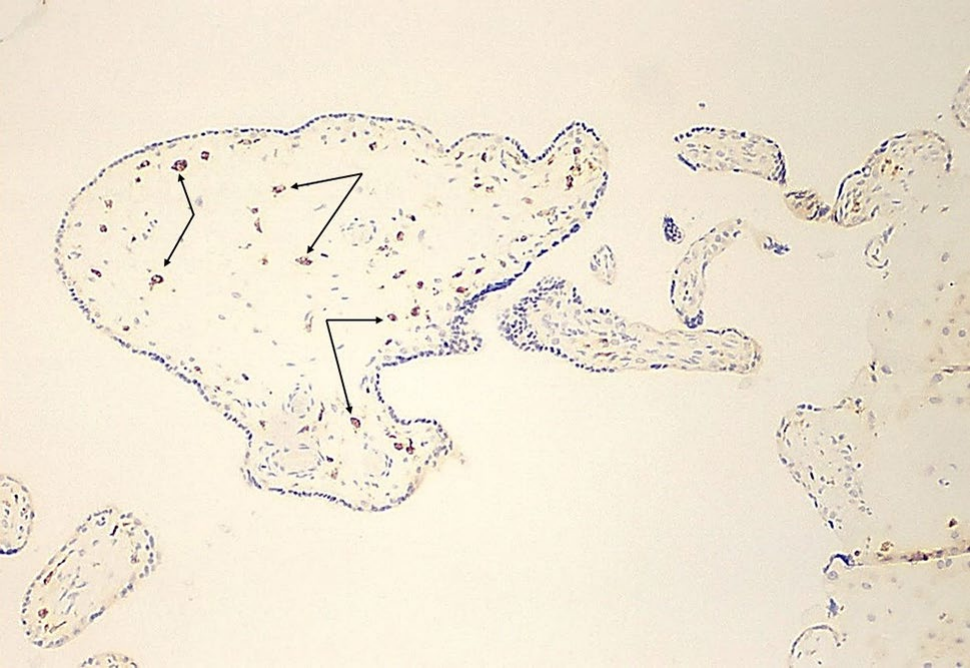
Chorionic villi from an uninfected placenta at the same gestational age (21 weeks) and magnification as shown in Fig. 4 using an antibody to macrophages, illustrating the normal number of Hofbauer cells (*arrows*) in the villous stroma

**Fig. 4:**
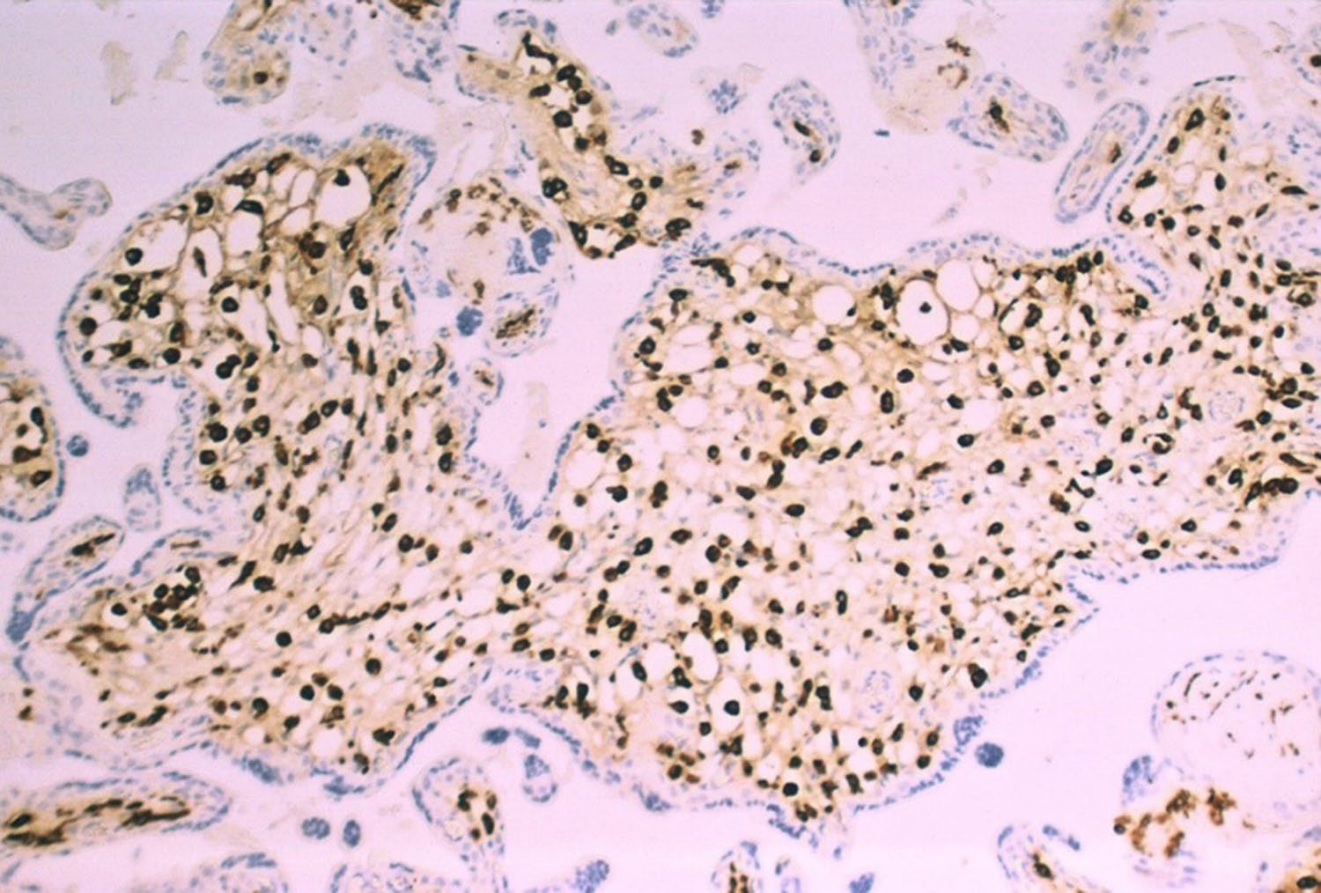
Abnormally increased numbers of Hofbauer cells can be identified in this enlarged chorionic villus from a 21 week gestation fetus using immunohistochemistry with CD-163 antibody. All the brown-staining cells in this image represent the nuclei villous stromal macrophages (Hofbauer cells). The fetus had microcephaly and Zika virus infection confirmed by RT-PCR

**Fig. 5:**
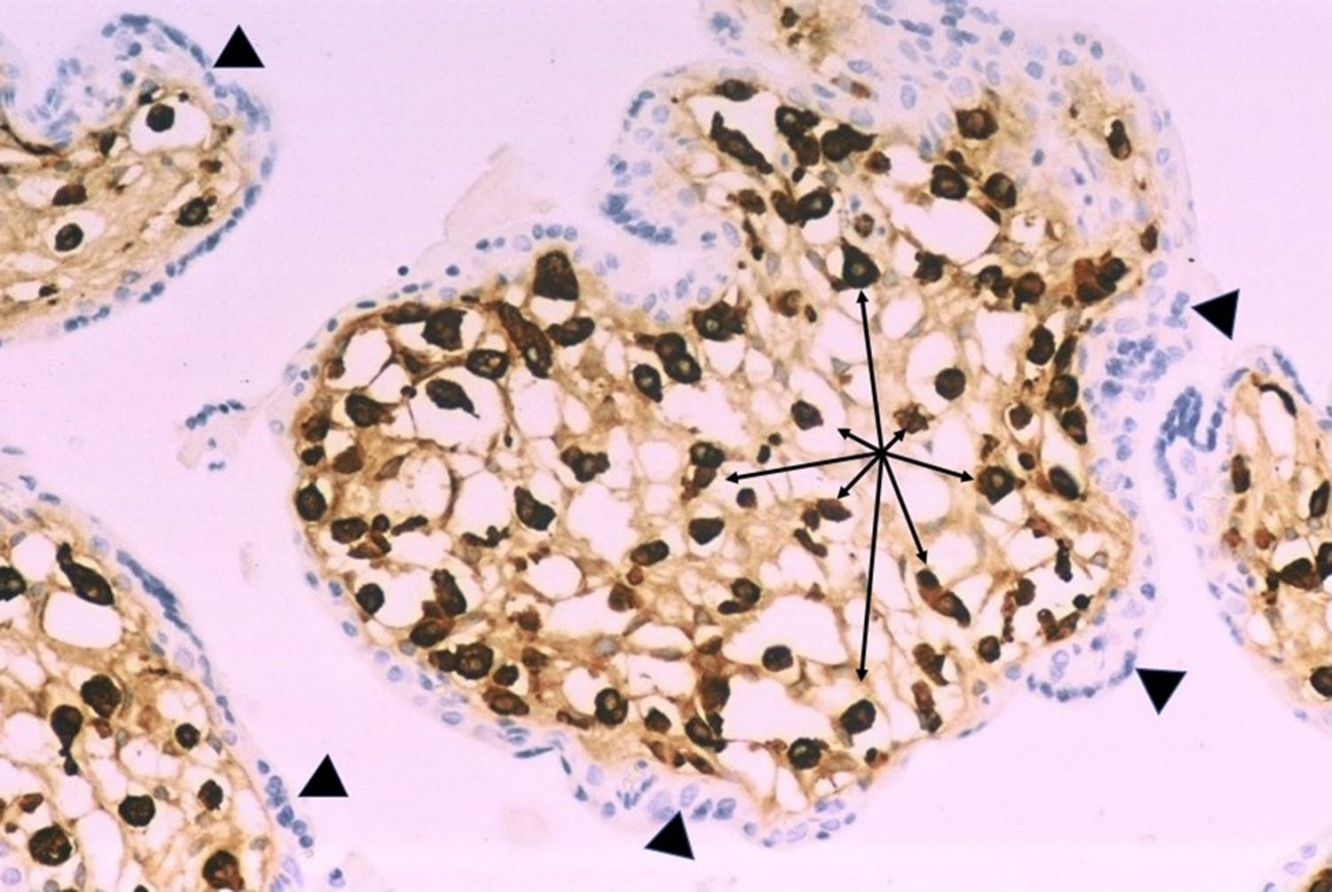
Higher magnification of Hofbauer cell hyperplasia in the terminal chorionic villi of a placenta from a 21 week gestation infant with intrauterine Zika virus transplacental infection and microcephaly. CD163 antibody. All the brown-staining structures (*arrows*) are nuclei of Hofbauer cells, which are tightly packed in the villous stroma. The trophoblastic outer layer (*arrowheads*) of the villi stains blue

Review of the second and third trimester placentas from fetuses with congenital Zika syndrome using immunohistochemistry for nuclear proliferation markers has revealed that Hofbauer cells undergo proliferation within the chorionic villous stroma in response to Zika virus infection of the placenta (Fig. 2). As a result, hyperplasia of Hofbauer cells develops within the chorionic villi which can be demonstrated using antibodies to macrophages such as CD68 or CD 163 (Figs. 3, 4, 5). This recent recognition of proliferation and hyperplasia of Hofbauer cells in response to hematogenous Zika virus infection is a potentially significant finding in aiding our understanding of the mechanism(s) of transplacental transmission of this virus [5, 20].

### Zika virus infects Hofbauer cells from placentas of human fetuses with microcephaly

**Fig. 6:**
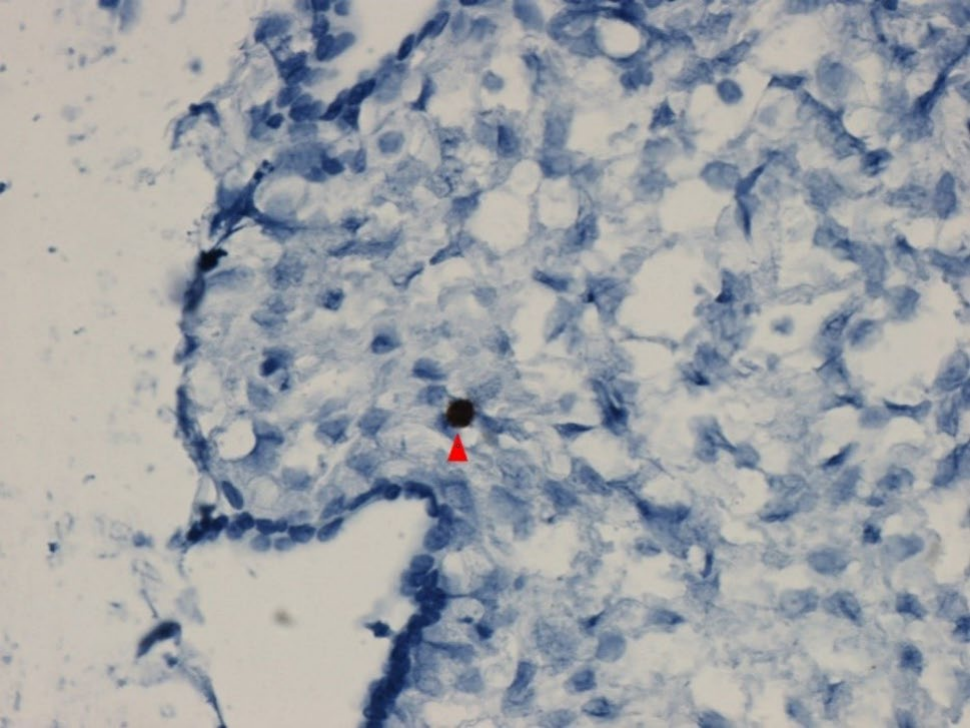
Zika virus RNA (*red arrow*) is positive in a stromal cell, presumably a Hofbauer cell, in the chorionic villus of the placenta of a 21 week gestational age fetus with congenital Zika virus infection and microcephaly. RNAscope

The Zika virus has recently been definitively identified in Hofbauer cells from placentas of human fetuses with intrauterine infection and microcephaly. Investigators have used RNA techniques to detect Zika virus nucleic acid [20] (Fig. 6) and antibody-based immunohistochemical methods to detect Zika viral antigen [5] in Hofbauer cells from placentas which were positive for Zika infection using reverse transcription-polymerase chain reaction (RT-PCR). Zika virus has been identified in the placental stromal Hofbauer cells for up to 10 weeks following initial maternal infection, indicating that the virus can persist in the placenta for long periods of time following the onset of maternal symptomatology [20].

### Zika virus has a wider range of placental cell tropism in first trimester placentas than later in gestation

In placentas examined from first trimester pregnancies, Zika virus has a much wider cellular distribution than in second and third trimester placentas. Zika virus has been identified in first trimester placentas of infected fetuses using immunohistochemistry in Hofbauer cells as well as in fetal leukocytes and endothelial cells [5]. In a study of 12 women infected with Zika virus who had miscarriage or spontaneous abortions occurring at less than 19 weeks’ gestation, replicative Zika virus RNA was identified in Hofbauer cells in nine cases, suggesting that these cells were directly infected early in gestation [21]. Although no chorioamnionitis was identified in these early gestation specimens, one placenta from a spontaneous abortion demonstrated chronic inflammatory cells on the maternal side of the placenta. Interestingly, no other placental cell types except for Hofbauer cells were observed to be infected in these nine cases.

Experimental infections using ex vivo first trimester placental organ and cell culture models have demonstrated Zika virus infection and replication in Hofbauer cells, mesenchymal cells and fibroblasts, and cytotrophoblast. Mesenchymal cells of the umbilical cord and cells from the decidua basalis including decidual fibroblasts and macrophages could also become infected [22]. The Zika virus has also been demonstrated to infect and replicate in first trimester and midgestational human maternal decidual cells grown ex vivo as three-dimensional (3D) organ cultures [23]. A recent report described the productive infection by two Zika virus strains of primary human endometrial stromal cells stimulated in vitro to undergo decidualization [24]. Tabata et al. have found that the Zika virus results in productive infection of Hofbauer cells as well as proliferating and invasive cytotrophoblast in human-derived first trimester villous explants [25]. In addition, they found that the Zika virus-infected primary human placental cells including cytotrophoblasts, endothelial cells, fibroblasts, and Hofbauer cells in chorionic villi, as well as amniotic epithelial cells and trophoblast progenitors in amniochorionic membranes that expressed AXL, TYRO3, and/or TIM1 viral entry cofactors. Based upon their data, these investigators suggested that there are two distinct potential pathways of early gestation vertical transmission of the Zika virus, including both placental and paraplacental routes.

### Zika virus infects and replicates in Hofbauer cells in experimental studies of human placentas

Zika virus has been found to be capable of infecting and replicating within Hofbauer cells from human term placentas [26, 27]. In one study, there was a 3-log increase in intracellular Zika virus RNA by 48 h following experimental infection of isolated human Hofbauer cells in vivo [27]. The virus which was shed from the Hofbauer cell was shown to be infectious to other cells. Experimental infection ex vivo of human placental explants with Zika virus has confirmed infection of villous stromal macrophages [25]. These experimental studies of Hofbauer cell infection corroborate the observations of Hofbauer cell infection by the Zika virus in human placentas from infants with congenital Zika syndrome.

### Trophoblast shows resistance to experimental Zika virus infection in some studies, but in other studies is permissive to virus replication

The syncytiotrophoblast of the placenta covers the chorionic villi, separating it from the surrounding maternal blood in the intervillous space. Lying immediately beneath these cells is the mononuclear cytotrophoblast, which among other functions, coalesces to form the multinucleated syncytiotrophoblast. Several experimental laboratory studies have provided evidence that both the syncytiotrophoblast and cytotrophoblast are resistant to Zika virus infection [26, 28]. However, in one investigation, Zika virus was shown to replicate in human cytotrophoblast cells, but to a lesser extent than in Hofbauer cells [24]. Recently, Sheridan et al. [29] found that cytotrophoblast and syncytiotrophoblast derived from placental villi at term resist in vitro infection by the Zika virus—these cells do not express genes encoding for most attachment factors implicated in ZIKV entry, but do express many genes associated with antiviral defense. In contrast, embryonic stem-cell-derived trophoblast, and particularly areas of syncytiotrophoblast within the colonies, quickly become infected and produced infectious Zika virus—these cells possessed numerous attachment factors for ZIKV entry, such as AXL and TYRO3, and lacked components of a robust antiviral response system.

## Discussion

Placental pathology has proven valuable in situations where infections may be transmitted from pregnant women to their fetuses, providing clinically relevant information on the mechanism(s) and timing of maternal-fetal (vertical) transmission, the nature of the inflammatory response by the mother and fetus to the agent, anatomic distribution and cellular localization of the agent, and the effects of the organism on the placental tissues and the fetal circulation, such as necrosis, hemorrhage or vascular disease. Unfortunately, so far during this current Zika virus pandemic, there have been relatively few placentas from the endemic countries that have been scientifically examined and published. However, it is not surprising that only a small number of placentas have been analyzed. During the recent 2013–2015 Ebola virus epidemic in West Africa, placental analysis would have been very useful in understanding the pathophysiology of disease transmission and potential methods for clinical intervention. Unfortunately, to date, there have been no published reports of placental pathology from Ebola virus-infected mothers and their infants from this epidemic. Even during the global human immunodeficiency virus (HIV) pandemic which was initially recognized in adult men in the US in 1981, and subsequently, in infants of infected mothers in 1982, it took greater than one decade for large case-controlled studies of human placentas from women and infants with and without HIV infection to be designed, implemented and analyzed [30–32].

Our analysis of the second and third trimester placentas from fetuses with congenital Zika virus infections does not demonstrate any significant inflammatory abnormalities, either of maternal or fetal origin. The absence of villitis, intervillositis, chorioamnionitis, and funisitis is unlike most other TORCH infections, which cause a spectrum of inflammatory abnormalities in the chorionic villi, umbilical cord, and placental membranes. It is important to communicate these observation of a lack of a maternal or fetal inflammatory response in placentas of Zika virus-infected fetuses from the second and third trimesters, because placental inflammation has been hypothesized by multiple authors in several articles as contributing to the development of placental dysfunction and insufficiency and the congenital Zika syndrome [33–35]—our findings would seem to negate these theories.

Some placentas from fetuses with congenital Zika syndrome have been described as containing microcalcifications and fibrin deposits. Microcalcifications within the placenta are non-specific findings which are considered to be normal, and occur in non-diseased placentas from fetuses with no underlying pathological processes. Excessive microcalcifications can be found in placentas with necrotizing villitis, cytomegalovirus infections, other causes of villous necrosis, maternal floor infarction, and massive perivillous fibrin deposition; however, Zika virus-infected placentas do not exhibit these tissue-destructive or ischemic processes. Focal or “patchy” fibrin deposition in placentas is also a normal finding unless excessive; however, there have been no reports of excessive fibrin accumulations in Zika virus-infected placentas. Both microcalcifications and fibrin deposition can be seen in placentas from stillborn fetuses from any cause, and are not specific for fetal demise resulting from Zika virus infection [5].

It has been approximately 12 months since the announcement in April 2016 of the Zika virus as a cause of fetal infections and malformations [1], and in these early stages of the Zika virus pandemic, all placental pathology information is clinically important to determine the mechanisms of fetal infection and help devise strategies for the diagnosis, intervention, and potential treatment of infants with congenital Zika virus infection. The observations in this communication, together with recently published reports of placental pathology from cases of human infection and experimental studies of placenta-derived cells, establish that Hofbauer cells can become infected, undergo proliferation, expand in number, and persist for many weeks within the chorionic villi of placentas following maternal infection with Zika virus. In particular, the occurrence of Zika virus remaining in a hyperplastic population of villous stromal macrophages (Hofbauer cells) of the chorionic villi for many weeks  to months after the initiation of maternal infection is clinically relevant, as this may provide a source of latent virus for continued infection of the fetus, even following the potential future development of clinical treatment modalities. Although the exact role of Hofbauer cells in facilitating the transplacental transmission of Zika virus is currently unknown, the pathology findings from human placentas in this report and others, taken together with mounting evidence obtained from experimental laboratory studies, strongly suggest an important or even primary role for these villous macrophages in fetal infection.
